# Dual Adhesion Pathways and Mechanotransduction of Adipose-Derived Mesenchymal Stem Cells on Glycated Collagen Substrates—Morphological Evidence

**DOI:** 10.3390/polym17243275

**Published:** 2025-12-10

**Authors:** Regina Komsa-Penkova, Borislav Dimitrov, Violina Ivanova, Svetoslava Stoycheva, Petar Temnishki, Konstantin Balashev, George Altankov

**Affiliations:** 1 Department of Biochemistry, Medical University Pleven, 5800 Pleven, Bulgaria; regina.komsa-penkova@mu-pleven.bg (R.K.-P.);; 2 Leonardo da Vinci Center of Competence in Personalized Medicine, 3D and Telemedicine, Robotic and Minimally Invasive Surgery, 5800 Pleven, Bulgaria; 3 Department of Physical Chemistry, Faculty of Chemistry and Pharmacy, University of Sofia, 1 James Bourchier Blvd., 1164 Sofia, Bulgaria; 4 Research Institute, Medical University Pleven, 5800 Pleven, Bulgaria

**Keywords:** MSC, collagen glycation, focal adhesion, mechanotransduction, YAP TAZ, RAGEs

## Abstract

Glycation-induced modifications of extracellular matrix (ECM) proteins, including collagen, are increasingly recognized as critical modulators of cellular behavior, particularly in pathophysiological contexts such as aging and diabetes. While their impact on general cell adhesion has been explored, the specific consequences for mesenchymal stem cell (MSC) mechanotransduction remain poorly defined. In this study, we investigated the temporal and mechanistic aspects of adhesion and mechanosensitive signaling in adipose-derived MSCs (ADMSCs) cultured on native versus glycated collagen substrates. Our findings identify two temporally distinct adhesion mechanisms: an initial pathway mediated by the receptor for advanced glycation end-products (RAGE), which is activated within the first 30 min following substrate engagement, and a later-stage adhesion process predominantly governed by integrins. Immunofluorescence analysis demonstrated maximal nuclear localization of YAP/TAZ transcriptional regulators during the initial adhesion phase, coinciding with RAGE engagement. This nuclear enrichment was progressively attenuated as integrin-mediated focal adhesions matured, suggesting a dynamic shift in receptor usage and mechanotransductive signaling. Interestingly, glycated collagen substrates accelerated early cell attachment but impaired focal adhesion maturation, suggesting a disruption in integrin engagement. Endogenous collagen synthesis was consistently detected at all examined time points (30 min, 2 h, and 5 h), suggesting a constitutive biosynthetic activity that remains sensitive to the glycation state of the substrate. Atomic force microscopy (AFM) demonstrated that glycation disrupts collagen fibrillogenesis: while native collagen forms a well-organized network of long, interconnected fibrils, GL-1 substrates (glycated for 1 day) displayed sparse and disordered fibrillary structures, whereas GL-5 substrates (5-day glycation) exhibited partial restoration of fibrillar organization. These matrix alterations were closely associated with changes in adhesion kinetics and mechanotransduction profiles. Taken together, our findings demonstrate that collagen glycation modulates both adhesion dynamics and mechanosensitive signaling of MSCs through a dual-receptor mechanism. These insights have significant implications for the design of regenerative therapies targeting aged or metabolically compromised tissues, where ECM glycation is prevalent.

## 1. Introduction

Cellular behavior is dynamically regulated through the integration of biochemical and biophysical signals originating from the surrounding microenvironment, which includes both the extracellular matrix (ECM) and neighboring cells [1]. Beyond metabolic and humoral factors—which are outside the scope of this study—cells possess specialized mechanosensitive receptors that detect and transduce mechanical cues into intracellular signaling cascades, ultimately leading to phenotypic adaptations. This process, termed *mechanotransduction*, is fundamental to a wide array of physiological and pathological processes, including embryogenesis, tissue homeostasis, aging, wound healing, and tumorigenesis [2,3,4].

Among the cell types most responsive to mechanical stimuli are mesenchymal stem cells (MSCs), whose fate decisions and regenerative capacity are tightly governed by mechanotransductive signaling [2,5]. Central to this mechanosensory machinery are integrins—transmembrane receptors that mediate cell–ECM adhesion and simultaneously sense matrix stiffness, topography, and other mechanical properties. These interactions orchestrate cytoskeletal remodeling and drive morphological and functional changes [6,7]. In addition to integrins, other adhesive receptors, such as members of the immunoglobulin superfamily (IgSF), may also modulate MSC behavior and contribute to their mechanosensitivity [8,9,10].

Collagen, the predominant structural protein within the ECM, plays a critical role in defining the matrix’s mechanical landscape, including its stiffness, porosity, and surface architecture [11]. These biomechanical attributes facilitate force transmission and regulate intercellular communication, thereby influencing diverse cellular functions [12,13,14,15]. Despite significant advances in ECM biology, the mechanistic implications of collagen-cell interactions—particularly under pathological conditions—remain incompletely elucidated [16,17].

One such pathological alteration is collagen glycation, a non-enzymatic modification driven by sustained hyperglycemia. This process involves the covalent attachment of reducing sugars to free amino groups in collagen, initiating the Maillard reaction and leading to the formation of early Amadori products [16]. Over time, these products evolve into advanced glycation end products (AGEs), which disrupt collagen’s supramolecular organization and alter its biomechanical properties [15,17]. In chronic diabetic conditions, excessive glycation compromises ECM integrity and perturbs mechanotransduction pathways, potentially impairing stem cell-mediated tissue regeneration and remodeling [18].

Mesenchymal stem cells (MSCs) possess the capacity to detect and respond to extracellular collagen through multiple mechanisms, with integrins playing a central role in mediating physical anchorage to the ECM and directing lineage specification and functional behavior [19,20]. These cell–matrix interactions are predominantly orchestrated through focal adhesions (FAs)—dynamic multiprotein complexes that serve as key mechanotransductive nodes, bridging ECM components, integrin receptors, and the intracellular cytoskeleton [21]. FA formation is particularly efficient on two-dimensional (2D) substrates, where biophysical parameters such as stiffness, surface topography, and surface energy critically influence their maturation and stability [11,21]. Moreover, the nanoscale spatial distribution of adsorbed adhesive proteins significantly modulates FA architecture and longevity [21,22]. Among the integrin heterodimers implicated in collagen recognition are α1β1, α2β1, and α6β1, which facilitate cellular adhesion and initiate downstream mechanotransductive signaling. Mechanical forces transmitted through integrins induce cytoskeletal remodeling and activate intracellular signaling cascades, notably the RhoA pathway. This involves the regulation of guanine nucleotide exchange factors (GEFs) and GTPase-activating proteins (GAPs), which modulate actomyosin contractility and cellular tension [23]. The dynamic reciprocity between collagen-integrin engagement and cytoskeletal signaling is essential for maintaining cellular morphology, polarity, and functional integration within the ECM. Through extracellular-signal-regulated kinase (ERK) and other pathways, integrin signaling affects genes involved in cell cycle progression, differentiation, and migration. Thus, mechanical signals from integrins can influence chromatin accessibility and histone modifications, linking ECM stiffness to transcriptional programs [24].

Recent insights have highlighted the Hippo signaling pathway as another critical downstream effector of mechanical stimuli, acting as a conduit for transducing extracellular mechanical cues into nuclear transcriptional responses [25,26,27]. This evolutionarily conserved pathway, originally characterized in *Drosophila melanogaster*, governs key biological processes including cell proliferation, survival, differentiation, and organ size regulation [28]. The Hippo pathway operates through a hierarchical kinase cascade involving the serine/threonine kinases STK3 (MST2) and STK4 (MST1), which form a complex with the scaffold protein Salvador (SAV1). This complex phosphorylates and activates the Large Tumor Suppressor Kinases (LATS1 and LATS2), which subsequently inhibit the transcriptional coactivators Yes-associated protein (YAP1) and transcriptional coactivator with PDZ-binding motif (TAZ) by promoting their cytoplasmic retention and degradation [26,29]. Through this mechanism, the Hippo pathway integrates mechanical inputs to regulate gene expression programs essential for tissue homeostasis and regenerative potential.

In our recent investigations, we provided both morphological and quantitative morphometric evidence of altered mechanotransductive signaling in adipose-derived mesenchymal stem cells (ADMSCs) adhering to collagen under oxidative stress conditions. These alterations implicated focal adhesions, actin cytoskeletal organization, and YAP/TAZ activity as key mechanistic components [15]. Furthermore, we demonstrated that early-stage glycation of collagen induces significant structural and biomechanical modifications, affecting FA assembly, integrin clustering, cytoskeletal dynamics, and the remodeling of the collagen matrix [15,17], as well as the kinetics of cellular attachment [10].

Building upon these findings, the present study aims to elucidate how post-translational modifications of collagen—specifically glycation—impact mechanotransduction in MSCs. Given the central role of ECM mechanics in stem cell fate determination, understanding these interactions is critical for advancing regenerative strategies under pathological conditions such as diabetes.

## 2. Materials and Methods

### 2.1. Collagen Procedures

#### 2.1.1. Collagen Preparation

As previously mentioned, rat tail tendon was extracted using acetic acid and then salted off with NaCl to produce almost pure collagen type I [30]. After centrifuging for 30 min at 4000 rpm and 4 °C, the pellets were redissolved in 0.05 M acetic acid and dialyzed versus 0.05 M acetic acid to get rid of the extra NaCl. As a result, a roughly monomolecular composition of rat tail collagen (RTC) solution was made, where the collagen concentration was close to 100% of the dry mass. Collagen isolation procedures were carried out at 4 °C. The modified Lowry test [31] and optical absorbance at 220–230 nm were used to determine the collagen concentration in the solutions [32].

#### 2.1.2. Preparation of Glycated Collagen

Rat tail tendon collagen type I (2 mg/mL) was pre-glycated by incubation in 500 mM glucose (Merck, Rahway, NJ, USA) in PBS at pH 7.4 with 0.02% NaN3 for one day and five days at 37 °C, as previously reported [18]. After dialysis against 0.05 M acetic acid, the samples were labeled RTC GL1 and RTC GL5, respectively, and stored in a refrigerator at 4 °C until needed.

### 2.2. Cells

Tissue Bank BulGen (Sofia, Bulgaria) provided human adipose tissue-derived mesenchymal stem cells (ADMSC) of passage two. The cells were obtained by liposuction with the prior written consent of regular donors. The cells were kept in DMEM/F12 media that was supplied by Sigma Aldrich (St. Louis, MO, USA) and included 10% fetal bovine serum (FBS), 1% GlutaMAXTM, and 1% antibiotic-antimycotic solution. The cells were employed in the tests up until the seventh passage, and the media was changed every two days until the cells attained approximately 90% confluency. Trypan blue exclusion testing was used to confirm ADMSC viability at the time of collection for each experiment. For every experiment, more than 85% vitality was considered acceptable.

#### 2.2.1. Morphological Studies

Regular glass coverslips (12 × 12 mm, ISOLAB Laborgeräte GmbH, Eschau, Germany) were coated with collagen (100 μg/mL) in 0.05 M acetic acid for the morphological observations. The coverslips were then put on 6-well TC plates (Sensoplate, Greiner Bioone, Meckenheim, Germany) and incubated for 60 min at 37 °C. Following three PBS washes, the cells were seeded in a final volume of 3 mL serum-free media at a density of 5 × 104 cells/well and incubated for 30 min, 2 h, and 5 h. For all morphological studies at the 2nd hour, 10% FBS was added. Using an inverted microscope (Leica Thunder Imager Live Cell, Leica Microsystems GmbH, Wetzlar, Germany) and phase contrast at magnification 20×, the initial cell adhesion and morphology were examined during the aforesaid time frame. The cells were then incubated before being prepared for immunofluorescent staining and morphometric analysis in two protocols, as follows:

##### First Protocol (Cell Spreading and FA Formation)

The triplet samples were fixed with 4% paraformaldehyde and permeabilized with 0.5% Triton X-100 after being incubated for 30 min, 2 h, or 5 h (at the 2nd hour, we added 10% serum). This was followed by saturation with FBS and further fluorescence labeling. The actin cytoskeleton was seen with green fluorescent Alexa FluorTM 488 Phalloidin (Invitrogen, Thermo Fisher Scientific Inc., Branchburg, NJ, USA), and the cell nuclei were stained with Hoechst 33342 (dilution 1:2000) (Sigma-Aldrich/Merck KGaA, Darmstadt, Germany).

Mouse Anti-Vinculin Monoclonal Antibody (Clone: hVIN-1, Thermo Fisher Scientific, Waltham, MA, USA) IgG (1:150) and fluorescent Alexa Fluor 555 conjugated goat anti-mouse IgG (minimal x-reactivity) antibody (supplied by Sigma-Aldrich) at a dilution of 1:100 were used to view focal adhesions.

##### Second Protocol (YAP/TAZ Signaling Events)

Separate samples from the same series were stained with a rabbit polyclonal anti-TAZ antibody and red fluorescent Alexa FluorTM 555 goat anti-rabbit antibody (both supplied by Sigma-Aldrich) in dilution 1:100 to track the YAP/TAZ signaling events. The samples were then counterstained with Hoechst 33342 (to view nuclei) and green fluorescent Phalloidin (Sigma-Aldrich, Darmstadt, Germany). Ultimately, all samples were placed upside down on glass slides using Mowiol and examined using a 20× objective on a Leica Thunder Imager Live Cell.

The CellProfiler 3.0 software was utilized to quantify the overall cell shape [33,34]. For every sample, at least three representative images were acquired. The various colors were combined with the corresponding image processing program. Every experiment was conducted four times. The distribution of a YAP/TAZ dataset was shown using box-and-whisker plots.

##### Overall Cell Morphology and Focal Adhesion Formation

Prior to fluorescence staining, the samples were fixed with 4% paraformaldehyde and permeabilized with 0.5% Triton X-100 after 30 min and two or five hours of incubation (at the second hour we added 10% serum). The actin cytoskeleton was seen with green fluorescent Alexa FluorTM 488 Phalloidin (Invitrogen, Thermo Fisher Scientific Inc., Branchburg, NJ, USA). Hoechst 33342 (dilution 1:2000) (Sigma-Aldrich/Merck KGaA, Darmstadt, Germany) was used to stain the cell nuclei. Anti-Vinculin Mouse Monoclonal Antibody (Clone: hVIN-1, Thermo Fisher Scientific, Waltham, MA, USA) IgG in dilution 1:150 and fluorescent Alexa Fluor 555 conjugated goat anti-mouse IgG antibody in dilution 1:100 (both supplied by Sigma-Aldrich) were used to visualize focal adhesions.

##### Quantitative Morphometry Analysis of Raw Format Images by ImageJ

ImageJ 1.54g software, which offers a variety of processing and analysis methods, was used for all per-cell image analyses [35]. Raw-format photos of cells (at 20X magnification) from at least 3 images taken under identical conditions were used to quantify fluorescence intensity. The regions of interest (ROIs) were highlighted and artifacts eliminated using pixel-based treatments. The segmentation module employed a default black-and-white threshold. Four metrics were obtained by analyzing images of equal sizes (W: 1600 px/H: 200 px): cell spread area (CSA), cell shape index (SCI), aspect ratio (AR) and cell perimeter (P). Otsu’s intensity-based thresholding method was used to generate binary masks of 20× fluorescent actin in order to determine the individual cellular domains. Cellular masks were then used to calculate ADMSC SA, CSI, and AR. The CSI was calculated using the formula

CSI = 4π × A/P^2^

where A is the mean cell area, and P is the mean cell perimeter.

When using this measure, a line has a CSI value of either 0 (which denotes an extended polygon) or 1 (which denotes a circle). The ratio of the cell’s largest to smallest side was used to compute AR.

##### Quantification of Intracellular Collagen Content

ADMSCs (5 × 10^4^) were seeded on collagen coated 22 × 22 mm coverslips in 6-well tissue culture plates and cultured serum free for 30 min and 2 h; 10% FBS was added for cultures maintained up to 5 h to preserve cell functionality.

For collagen detection, cells were fixed with 4% paraformaldehyde and permeabilized using 0.5% Triton X-100. Non-specific binding sites were blocked with 5% bovine serum albumin (BSA). Samples were then incubated with a mouse monoclonal anti-human collagen type I antibody (Sigma Aldrich, 1:200 dilution) for 1 h at room temperature, followed by a green-fluorescent Alexa Fluor™ 488-conjugated anti-mouse secondary antibody (Invitrogen, Thermo Fisher Scientific Inc., Branchburg, NJ, USA). Nuclear counterstaining was performed using Hoechst 33342 (1:200 dilution; Sigma-Aldrich/Merck KGaA, Darmstadt, Germany). Fluorescence imaging was conducted using an inverted fluorescence microscope, Leica Thunder Imager, as above, and collagen expression was quantified with ImageJ software. Box-and-whisker plots were used to represent the dataset’s distribution of endogenous human collagen. Box-and-whisker plots were used to display a YAP/TAZ dataset’s distribution.

### 2.3. Atomic Force Microscopy of Glycated Collagen

#### 2.3.1. Sample Preparation

For Atomic Force Microscopy (AFM) imaging, a solution from native or glycated collagens (GL1 and GL5) was deposited onto freshly cleaved, square mica sheets (Structure Probe Inc./SPI Supplies, West Chester, PA, USA) that were adhered to metal pads. Approximately 100 µL of the collagen solution was applied to the mica support. After a 15 min incubation, the samples were washed with water, PBS, and water, thoroughly dried under a stream of nitrogen gas, and then transferred for AFM imaging.

#### 2.3.2. AFM Imaging

AFM imaging was performed using a NanoScope V system (Bruker Inc., Santa Barbara, CA, USA) in tapping mode under ambient air conditions. Standard silicon nitride (Si3N4) probe tips (BudgetSensors; Innovative Solutions Ltd., Sofia, Bulgaria) with a tip radius of less than 10 nm were used. Samples were scanned at a rate of 1 Hz. Each sample was examined at 3 to 5 randomly selected locations across the mica surface. Images (512 × 512 pixels) were acquired in both height and phase modes.

#### 2.3.3. Morphological Characterization

Morphological characterization, including the determination of height, spreading area, and average roughness of the films containing the collagen fibrils, was performed using Bruker NanoScope Analysis 1.3 software. Roughness analysis was conducted as described elsewhere [36].

### 2.4. Statistical Analysis

All experiments were performed in a minimum of three independent series for each group. Because the morphometric parameters did not follow a normal distribution (as verified by Levene’s test), comparisons between subgroups of cells adhering to native and glycated collagen were carried out using a rank-based one-way ANOVA (Kruskal–Wallis test), which is more suitable for this data type.

Differences among subgroups in NAT (RTC), GL1, and GL5 collagens were further analyzed using Dunn’s post hoc test.

Box-and-whisker plots, a visual tool that displays a dataset’s distribution using its five-number summary: minimum, first quartile (Q1), median (Q2), third quartile (Q3), and maximum, were used for a dataset of YAP TAZ and endogenous human collagen presentation. Significance is indicated by asterisks (*), corresponding to *p* < 0.05, or (**) corresponding to *p* < 0.01. visual tool that displays a dataset’s distribution using its five-number summary: minimum, first quartile (Q1), median (Q2), third quartile (Q3), and maximum.

## 3. Results

### 3.1. Morphological Response of Stem Cells on Glycated Collagen

**Figure 1:**
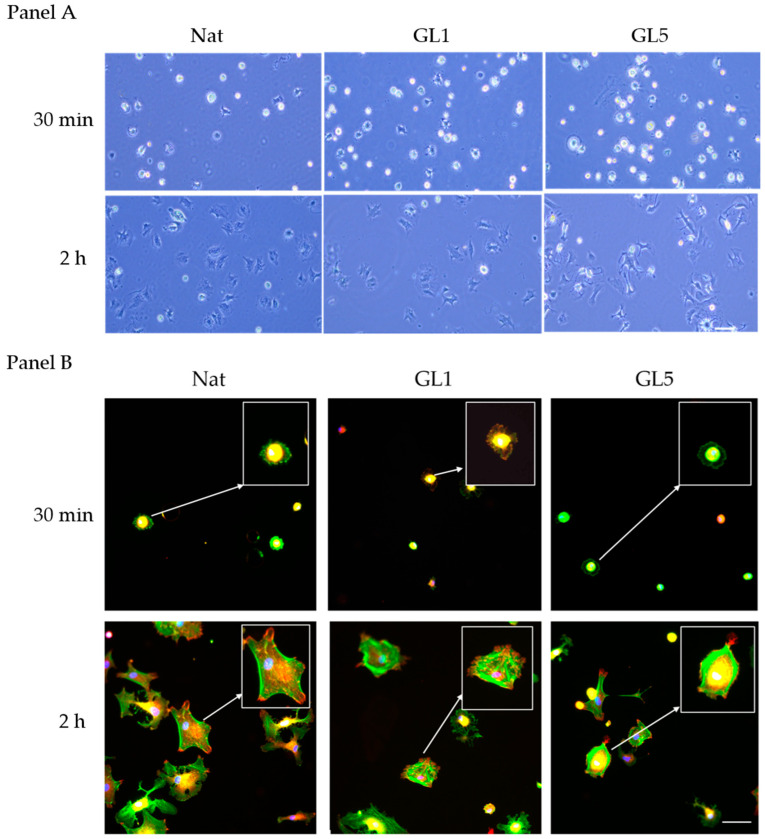
Overall Morphology of ADMSC adhering for 30 min and 2 h on glycated collagen. The cells were cultured for 30 min or 2 h on collagen coated glass slides coated with either native collagen NAT or glycated collagen for 1 day GL1 or 5 days GL5 ). On (**Panel A**) the cells are viewed directly from the plates under phase contrast, or fixed and further stained with FITC phalloidin (**Panel B**) to view the actin cytoskeleton (green) while nuclei are counterstained with Hoechst (blue). Arrows denote inserts with an enlarged view of the corresponding cell. Bar 20 μM.

As demonstrated by phase contrast images of living cells captured directly from the plate (Figure 1 Panel A), under static culture conditions, AD-MSCs exhibit efficient initial adhesion at 30 min (left row) and further spread well at the 2nd hour mark. The initial adhesion however significantly increases on glycated collagen samples, both Gl1 and Gl5, while tend to be reduced at the 2nd hour mark, an effect that was well documented previously [1,10,15,17], and now validated by our current findings. As shown in Figure 1 Panel B (bottom row), within two hours post-seeding, AD-MSCs exhibit a robust organization of the actin cytoskeleton, characterized by extensive formation of stress fibers (Figure 1 Panel B (Nat, 2 h, viewed in green), which coincide with focal adhesion (FA) complexes (red), better seen on the augmented 2× insert, and also below. In contrast, collagen glycation markedly impairs cellular spreading activity. Notably, substrates subjected to five days of glycation (GL5) induce significant cellular shrinkage (Figure 1, Panel B, GL5 for 2 h), suggesting an altered substratum interaction, whereas those glycated for one day (GL1) promote an intermediate morphological phenotype (Figure 1, Panel B, GL1 for 2 h). These observations are consistent with our earlier study, supporting a hypothesis of altered integrin-mediated recognition of glycated collagen [10,15,17].

An inverse trend was observed when ADMSC adhesive interactions were assessed at the early 30 min incubation stage (Figure 1, Panel B, upper row). At this time point, cells exhibited visibly enhanced attachment to glycated collagen substrates (Figure 1 Panel B upper row) compared to native collagen (Figure 1, Panel B, first left image).

**Figure 2:**
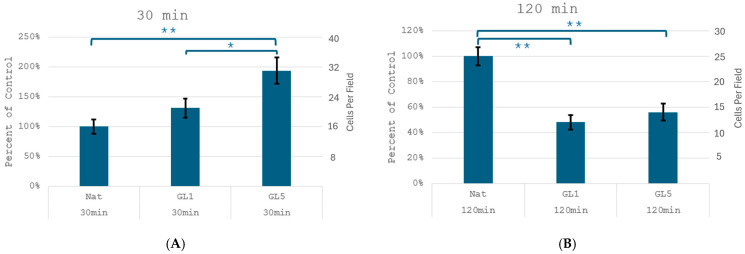
Quantification of cell adhesion dynamics. The cells were cultured for 30 min (**A**) and 2 h (**B**), on the above collagen substrata, either native (Nat), or glycated GL1 and GL5, then fixed and stained with Hoechst before counting the nuclei in 10 randomly chosen fields. Data are expressed as mean ± standard deviation (SD), with the number of biological or technical replicates indicated as n. Statistical significance is denoted by asterisks: *p* < 0.05 (*), and *p* < 0.01 (**).

These morphological observations corroborate the direct quantities of cell adhesion (Figure 2), which revealed a significant increase in the number of adherent cells at the 30 min mark on glycated samples—from 15 ± 2 cells/field on native collagen (Nat) it increases to 21 ± 3 and 32 ± 4 cells/field on GL 1 and GL 5, respectively. For clarity these results are also presented as % increase vs. control (left axis), showing about 140 and 190% increase for Gl1 and Gl5, respectively. Conversely, at 2nd hour mark adhesion significantly drops on glycated samples in about 45 and 48% for Gl1 and Gly5 samples, respectively, from about 25 ± 3 cells/slide to about 12 ± 1 and 14 ± 2 cells/slide, for GL 1 and GL 5, respectively. These quantities are in harmony with the morphological observation in Figure 1 at panels A and B.

**Figure 3:**
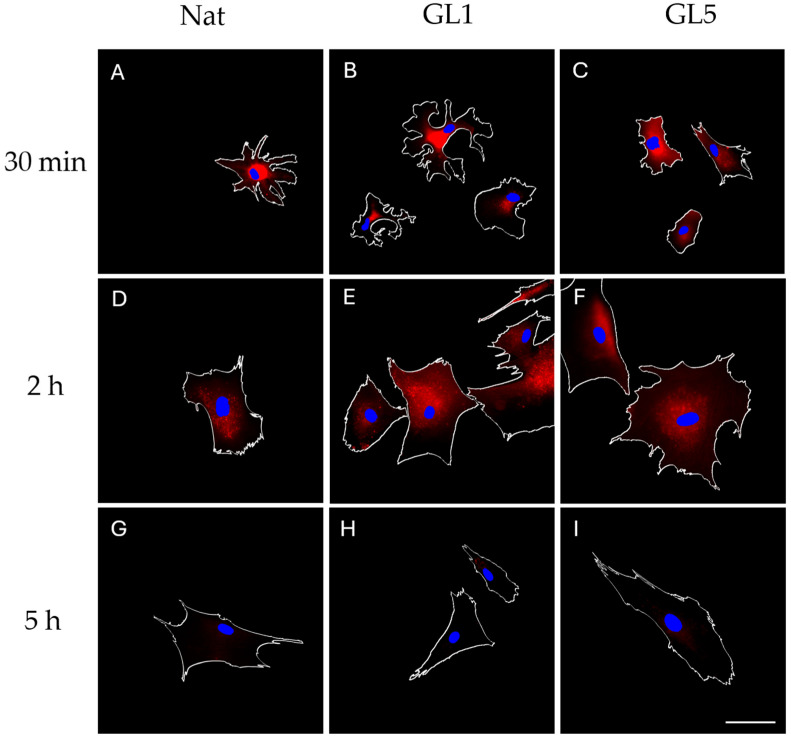
Immunofluorescence visualization of RAGE expression at early (30 min) and late (2 h and 5 h) time points. Mesenchymal stem cells were cultured on collagen-coated glass slides pre-treated with either native collagen (**A**,**D**,**G**), GL1-collagen glycated for 1 day (**B**,**E**,**H**), or GL5-collagen glycated for 5 days (**C**,**F**,**I**). Cells were maintained under serum-free conditions for 30 min and 2 h, while 10% FBS was added to the samples cultured for 5 h. Following incubation, cells were fixed and stained with rabbit polyclonal anti-RAGE antibody (red) and visualized using Alexa Fluor 555-conjugated goat anti-rabbit secondary antibody). Nuclei were counterstained with Hoechst (blue). Scale bar: 20 μm.

To address potential variability in morphological characteristics across distinct microscopic fields, ImageJ software 1.54g version (Windows, Mac, Linux) was employed in a follow-up experiment to quantitatively assess cell adhesion efficiency. Parameters analysed included mean cell spreading area (CSA, μm^2^), perimeter (μm), and cell shape index (CSI) of the cells presented in Figure 3. This experiment also incorporated an extended incubation period of 5 h. The resulting data are summarised in Table 1.

As shown in Table 1—and despite the apparently improved attachment observed in Figure 2—ADMSCs exhibit reduced adhesion efficiency to glycated collagen substrates during the initial 30 min of incubation. This is most evident in the cell spreading area (CSA), which decreases by approximately 58%, from 3165 μm^2^ on native collagen (Nat) to 1337 μm^2^ and 1326 μm^2^ on GL1 and GL5 substrates, respectively. A similar reduction is observed in cell perimeter, which declines by roughly 45%, from 225 μm on Nat to 144 μm and 136 μm on GL1 and GL5, respectively. Prolonged incubation for 5 h results in only a modest increase in perimeter (~20%), indicating limited enhancement of cell spreading over time. The cell shape index (CSI) however decreases progressively from 0.7 to 0.2 between 30 min and 5 h, reflecting a morphological transition from rounded to more elongated cell profiles. These findings suggest that although initial adhesion to glycated collagen appears accelerated (as illustrated in Figure 1 and Figure 2), it is characterized by restricted spreading and morphological adaptation, indicative of a rapid yet suboptimal adhesion process.

By 2 h of incubation, ADMSCs cultured on native collagen exhibit a pronounced enhancement in adhesion efficiency, reflected by an approximate twofold increase in both cell spreading area (CSA) and perimeter—from 3165 μm^2^ to 7522 μm^2^, and from 225 μm to 468 μm, respectively. In contrast, glycated collagen substrates continue to suppress these parameters by roughly 20%. At the 5-h mark, cells begin to partially restore their spreading capacity, as indicated by a modest increase in perimeter and a ~20% decrease in cell shape index (CSI), consistent with a shift toward a more elongated, adherent morphology.

This temporal pattern is consistent with our recent findings [10], demonstrating that ADMSCs rapidly adhere to glycated collagen substrates under both static and flow-based conditions, followed by a gradual decline in adhesion strength and cell spreading at later time points. These observations prompted the hypothesis that additional adhesive receptors may be involved in mediating early interactions with glycated matrices. As previously proposed [10], particular attention was given to the receptor for advanced glycation end products (RAGE), a member of the immunoglobulin superfamily (IgSF)—a broad class of cell surface and soluble proteins implicated in cellular recognition, binding, and adhesion processes [37,38].

Although our earlier experiments did not reveal substantial RAGE expression in ADMSCs, literature reports suggest that RAGE may be transiently expressed under specific conditions, including exposure to extrinsic microenvironmental cues and prolonged in vitro culture [39,40,41]. To address this, the present study undertook a more systematic morphological investigation of RAGE expression dynamics over extended incubation periods.

### 3.2. RAGE Expression in ADMSCs During Collagen Adhesion

RAGE expression was evaluated at 30 min, 2 h, and 5 h after ADMSC adhesion to collagen substrates. Up to 2 h, experiments were conducted under serum-free conditions to ensure specific collagen adhesion (integrin clusters already formed), after which 10% serum was added to maintain cell functionality.

Representative images in Figure 3A–F reveal relatively low levels of RAGE expression, primarily detectable during the early incubation intervals—namely at 30 min and 2 h. Notably, no discrete adhesive clusters were observed at the cell periphery. Instead, RAGE localization appeared diffusely distributed, concentrated rather in the cells middle, but not in the nucleus, suggesting potential association with intracellular compartments or extracellular deposition zones, such as the underlying substratum. By the 5 h time point, RAGE expression showed a pronounced reduction.

### 3.3. ADMSC Mechanotransduction from Glycated Collagen

Cell–matrix interactions are predominantly mediated by focal adhesions (FAs), which—as outlined in the introduction paragraph—serve as central hubs for mechanotransduction by linking ECM components to integrins and the actin cytoskeleton [21]. In parallel, emerging evidence underscores the pivotal role of the Hippo signaling pathway in relaying mechanical cues to the nucleus. To concurrently assess FA dynamics and Hippo pathway activation during ADMSC adhesion to glycated collagen substrates, we focused on the immunofluorescent visualization of FAs and the YAP/TAZ signaling axis, the principal intracellular effectors of Hippo pathway.

**Figure 4:**
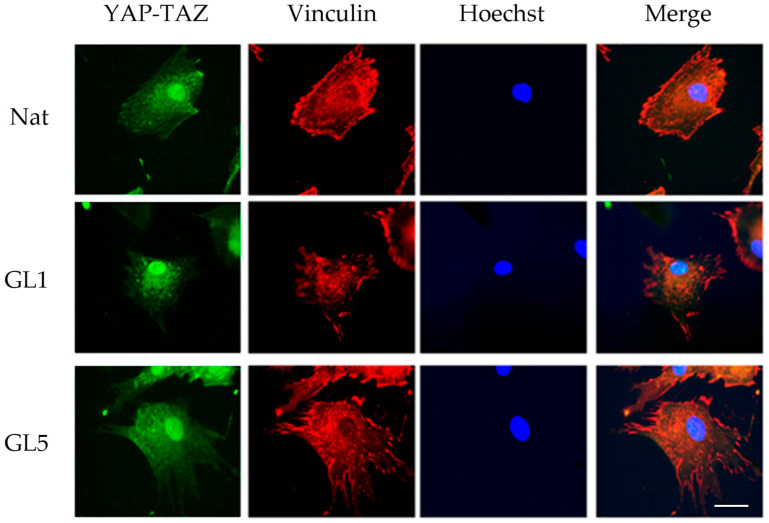
Simultaneous visualization of focal adhesion formation and YAP/TAZ activity of ADMSC on glycated collagens. ADMSC were cultured for 2 h on native or glycated collagen (for 1 day or 5 days) in serum-free medium, then stained for vinculin (red), YAP/TAZ (green) and nuclei (blue). Bar 20 µm.

A preliminary investigation was conducted using a previously validated protocol involving a 2 h incubation under serum-free conditions [17]. Following incubation, cells were fixed and subjected to dual immunostaining: anti-vinculin antibody was employed to visualize focal adhesions, while anti-TAZ antibody labeling was used to assess Hippo pathway activity (Figure 4). Quantification of TAZ activity was based on the ratio of nuclear to cytoplasmic signal intensity. To ensure accurate spatial resolution, nuclear counterstaining was performed, enabling clear delineation of intra- and extranuclear compartments for precise assessment of TAZ distribution.

The morphological data obtained from this study revealed several notable findings:

At this time point (2 h) of ADMSC incubation, collagen glycation led to a reduction in the number of FAs. Nevertheless, these structures remained detectable (red), showing a persistent presence despite the overall decline. However, a subtle and rather inconclusive trend was observed toward nuclear translocation of YAP/TAZ, suggesting a potential—but not definitive—activation of this pathway under our experimental conditions.

These observations underscored the need to implement a morphometric approach for the quantitative assessment of focal adhesion (FA) dynamics and YAP/TAZ activity in a time-resolved manner. This strategy enables the characterization of the temporal progression of these processes and facilitates the determination of their dependence on incubation duration.

#### 3.3.1. Quantification of Focal Adhesion (FA) Dynamics

**Figure 5:**
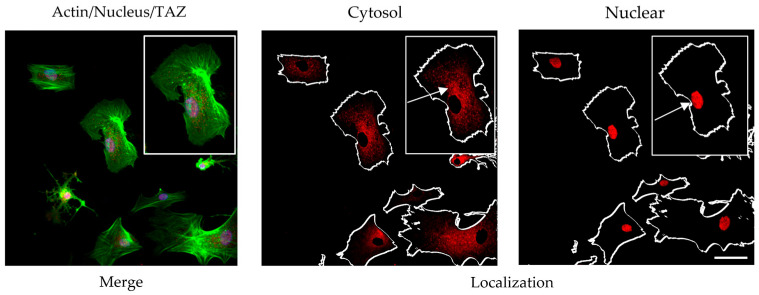
Quantification of YAP/TAZ activity in a pilot study. ADMSCs were cultured for 2 h on collagen-coated slides, then fixed and co-stained for actin (green) and nuclei (blue) as shown in the left panel. The actin (**left panel**) is visualized to get the contour of the cell. TAZ localization was visualized using an anti-TAZ antibody (red), highlighting cytoplasmic (**middle panel**) and nuclear (**right panel**) regions. The actin (**left panel**) is visualized to get the contour of the cell. The arrows on the right panel (Nuclear) indicate nuclear Taz localization. The arrows on the middle panel (Cytozol) indicate cytosolic YAP/TAZ localisation. Image analysis was performed using ImageJ 1.54g software. Bar 20 µm.

Data is presented in Table 2, which summarizes the morphometric results across all experimental settings, together with the number of cells analyzed. FA dynamics were quantified using ImageJ 1.54g software. As anticipated in Table 2, minimal FA formation was detected after 30 min of incubation, despite the improved initial adhesion observed on glycated substrates (Figure 1 and Table 1). At this early time point, the number of FAs per cell was negligible (ranging from 1 to 2 per cell). By the 2 h mark, FA formation markedly increased on native collagen, reaching 98 ± 29 FAs per cell. In contrast, FA numbers were significantly reduced on glycated collagen, with GL1 and GL5 showing approximately 40% and 35% decreases, respectively (Table 2, Figure 5). The same trend follows also the Total area of FA, showing about a two-fold drop for GL1 and about a triple reduction for GL5 (from an initial 1407 µm^2^ for Native collagen to 782 and 492 µm^2^ for GL1 and GL5, respectively.

#### 3.3.2. Quantification of YAP/TAZ Activity

The ImageJ software was further used to quantify the coordinated YAP/TAZ translocation to the nucleus, as exemplified in Figure 3:

For this preliminary experiment, actin was used to outline the overall cell shape as a region of interest (ROI) within the cytosol, while the ROI of the nucleus was marked from the blue channel (both shown with arrows). This allowed us to measure separately the fluorescence intensity, representing YAP/TAZ activity of the nucleus and the cytosol on the red channel, and providing opportunity their ratio (nucleus vs. cytosol) to be calculated.

**Figure 6:**
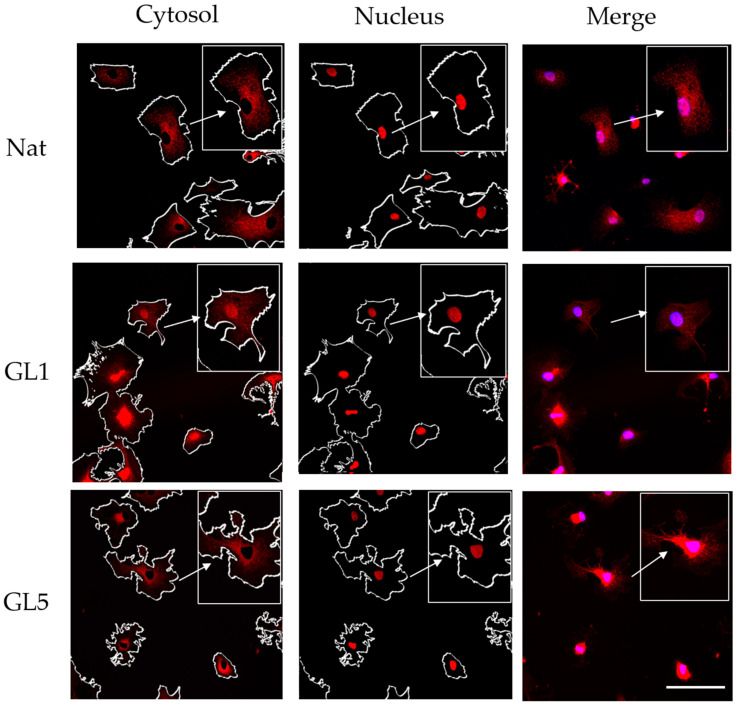
Overall characteristic and YAP/TAZ activity after 2 h incubation of ADMSC on native or glycated collagen, as indicated for 1 day (GL1) or 5 days (GL5). ADMSC were cultured serum-free for 2 h on native and glycated collagen, then fixed and stained with anti-TAZ antibody (red) and nucleus (blue), shown on the right panel (Merge), as detailed in Figure 3. Actin staining was used to delineate cell boundaries; however, the green channel was omitted to enhance clarity of the red YAP/TAZ distribution and the white contours. Arrows in the Nuclear panel indicate TAZ localization within the nucleus, while arrows in the Cytosol panel highlight YAP/TAZ localization in the cytoplasm. The Merge panel displays both localizations combined. ImageJ 1.54g software was used to outline the activity in the cytosol (left row) vs. the nuclear regions (middle row). Bar 20 μM.

The next experiment was already designed to compare the YAP/TAZ activity of ADMSC between native and glycated collagens, GL1 and GL5, respectively (Figure 6). Figure 4 presents the morphological view upon evaluation of YAP/TAZ activity at the 2nd hour of incubation.

**Figure 7:**
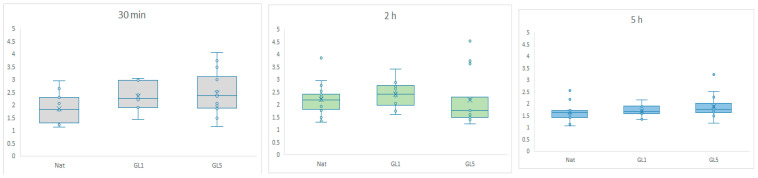
Dynamics of YAP/TAZ activity on glycated collagens at different time points.

To provide a comprehensive overview of the process, additional time points were analyzed and are presented in Figure 7 as quantitative data—expressed as nuclear-to-cytosolic fluorescence intensity ratios—covering 30 min, 2 h, and 5 h of incubation. Quantification was performed using ImageJ 1.54g.

The graphs depict YAP/TAZ nuclear accumulation, quantified as the ratio of fluorescence intensity between the nuclear region of interest (ROI) and the cytosolic ROI, as presented on the Y scale. Box-and-whisker plots illustrate the distribution of nuclear signaling efficiency, showing the median and interquartile range (IQR). Comparisons were performed across native collagen (control), GL-1 (1-day glycated), and GL-5 (5-day glycated) substrates at three time points: 30 min (gray), 2 h (light green), and 5 h (blue). A significant difference was found between native and GL5 at 30 min, GL1 at 30 min and 5 h, and GL5 at 30 min and 5 h.

Figure 7 summarizes the quantitative data, revealing a marked nuclear accumulation of YAP/TAZ during the early adhesion phase (30 min), with a particularly strong signal in cells cultured on glycated collagen substrates. This increase corresponds to elevated nuclear-to-cytoplasmic localization ratios, indicative of effective mechanotransduction. In glycated conditions—especially GL1—YAP/TAZ nuclear localization continues to intensify up to 2 h. In contrast, GL5 substrates show a declining signal beyond this point, which is largely resolved by 5 h. These findings suggest that glycated collagen induces a rapid, but rather transient activation of YAP/TAZ signaling, peaking during the initial 30 min adhesion window.

### 3.4. Morphological Evidence for an Increased Collagen Synthesis of ADMSC on Glycated Collagen

**Figure 8:**
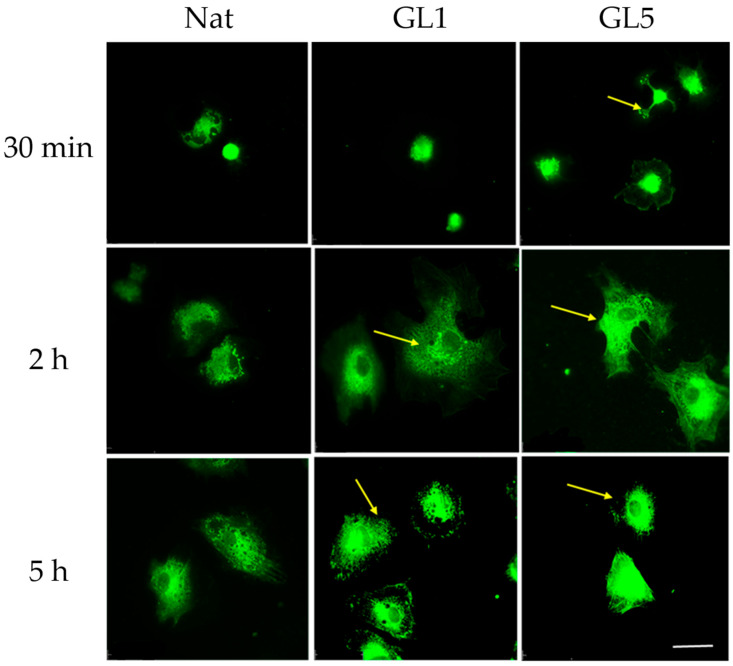
Human collagen type I synthesis by ADMSCs on native and glycated collagen substrates. ADMSCs were cultured for 30 min, 2 h, and 5 h (adding 10% serum at 2nd hour) on native collagen and glycated collagen matrices prepared for 1 day (GL1) or 5 days (GL5). Afterwards, the cells were fixed and stained with an anti-human collagen type I antibody, followed by a green fluorescent anti-rabbit secondary antibody. Arrows highlight the perinuclear accumulation of collagen-containing vesicles. Scale bar: 50 μm.

Although enhanced YAP/TAZ signaling was observed in ADMSCs at 30 min of adhesion to glycated collagen, this did not correspond to an immediate increase in collagen production, as evidenced by intracellular staining patterns shown in Figure 8. Beginning at the 30 min time point, ADMSCs exhibited a progressive increase in fluorescence intensity indicative of intracellular collagen content, primarily localized to the perinuclear region—suggestive of various stages of Golgi-mediated processing. Notably, by the 5 h mark, distinct secretory granules became apparent at the cell periphery (arrows, bottom panel), indicating active endogenous collagen secretion. These findings suggest that collagen synthesis and secretion may proceed independently of early YAP/TAZ activation.

**Figure 9:**
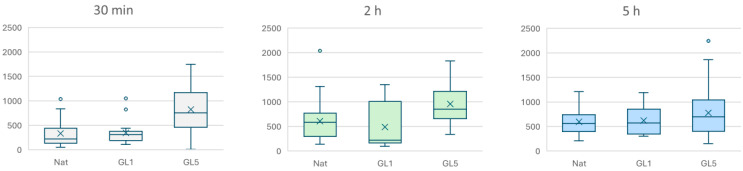
Quantification of the endogenous collagen fluorescence in individual ADMSCs.

To validate this trend, we further analyzed endogenous collagen content at the same time points of ADMSC adhesion to native and glycated collagen substrates. The graph in Figure 9 summarizes the data on the quantification of total collagen content per cell using a monoclonal antibody followed by a fluorescently labeled secondary antibody. Cells were incubated for 30 min, 2 h, and 5 h, as stated in the Figure 8, legend and collagen content was assessed via ImageJ 1.54g software.

ADMSCs were cultured under the same conditions as described in Figure 8. Following incubation, cells were fixed and immunostained using an anti-human collagen type I primary antibody, with visualization achieved via a green-fluorescent goat anti-rabbit secondary antibody. Fluorescence intensity, expressed as Arbitrary Photometric Units (APU) per cell, was quantified using ImageJ 1.54g software. Box-and-whisker plots represent the intensity of endogenous collagen fluorescence in AU (Arbitrary Units), displaying the median, interquartile range (IQR), and full data spread. Comparisons were made across native collagen (control), GL-1 (1-day glycated), and GL-5 (5-day glycated) substrates at three time points: 30 min (gray), 2 h (light green), and 5 h (blue).

As shown in Figure 9, the intensity of the endogenous collagen signal increases with prolonged incubation, in a comparable ratio under both native and glycated conditions, and appearing to peak around the 2 h mark. Notably, GL5 consistently displays higher fluorescence than GL1, with nearly double the signal at 30 min and a sustained, though attenuated, difference at 2 and 5 h.

### 3.5. AFM Studies of Glycated Collagen

**Figure 10:**
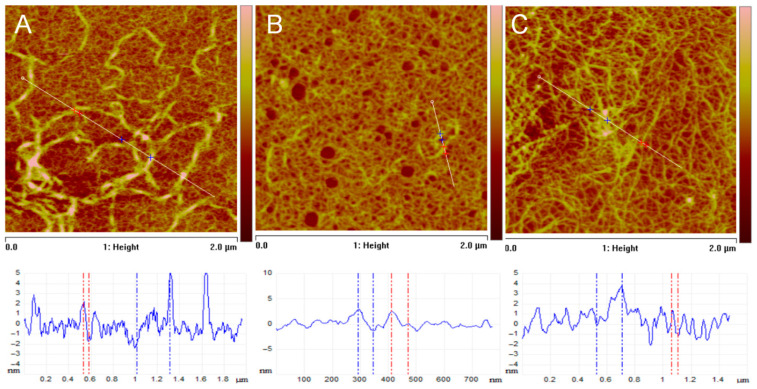
AFM images illustrating topographical changes and fibril formation in collagen upon glycation. (**A**) Native collagen, (**B**) GL1 modified collagen, (**C**) GL5 modified collagen. Each panel shows a 2D height image with corresponding cross-sectional profiles (in blue) in the cross-section plots taken along the indicated scan lines (white lines) on the 2D (**A**–**C**) Images. The red and blue dotted lines in the cross-section plots mark the specific points used to determine fibril dimensions, indicating where height (z-direction) and lateral width (xy-direction) were extracted. Images were acquired at a 2 × 2 µm scan size with a z-range of 10 nm, providing detailed morphological information and size distributions of the collagen fibrils.

Cell adhesion may strongly depend on the geometry of the underlying substratum and the positional cues that ADMSCs can recognize there, which can be relatively easily studied by Atomic Force Microscopy (AFM). Typical AFM images of collagen films deposited onto mica supports, obtained from solutions of native and glycated collagens, along with corresponding cross-sectional profiles, are presented in Figure 10. These images reveal strict differences in morphology, network formation, and fibril structure upon glycation of collagen.

Figure 10A depicts a typical self-assembled collagen network on mica, demonstrating the characteristic fibrillar morphology of native collagen. More specifically, for the morphology of the film from native collagen in Figure 10A, one can identify a well-defined network of long, interconnected fibrils. The fibrils are relatively uniform in width and appear to be well-assembled (Figure 10A). The height profile (bottom graph) reveals significant vertical variations, indicating distinct fibrils rising above the scanned surface. Considering the network density, it appears to be relatively dense, with good surface coverage, as evidenced by the surface roughness measurements, which yield average values of $R_{a}=1.0\;nm.$ Furthermore, section analysis, as shown in the profile of Figure 10A, indicates collagen fibril heights of 5 ±1 nm, and widths ranging from tens to hundreds of nanometers, consistent with typical collagen fibril sizes measured by AFM [42].

In Figure 10B, a representative film of GL1 glycated collagen on a mica substrate is shown, illustrating that glycation (GL1) significantly disrupts the self-assembly of collagen into well-defined fibrils. This suggests that glycation interferes with normal fibrillogenesis, likely by altering the protein’s structure and hindering proper intermolecular interactions. The morphology of the film reveals a much sparser distribution of collagen compared to native collagen (Figure 10A). During the self-assembly process on the mica surface, some regions remain uncovered, appearing as round, substrate-exposed areas. Additionally, there are fewer distinct fibrils, and the surface appears more aggregated and less organized. The height profile exhibits reduced variation, indicating a flatter surface with fewer prominent features. Indeed, the estimated surface roughness is nearly half that of the native collagen sample, with an Rₐ value of 0.6 nm. As seen in the image, the collagen network is sparse and poorly developed. Fibril dimension measurements further confirm that the fibrils are smaller and less defined than those in the native sample, with diameters ranging approximately from 1 to 2 nm.

Figure 10C clearly shows that glycation in the GL5 sample also disrupts collagen self-assembly, but perhaps to a lesser extent than in the GL1 sample. This suggests that the degree of glycation or the specific glycation sites might influence the extent of disruption in collagen fibrillogenesis [43]. Analyzing the morphology of the film, one can argue that a network is more developed than that of the GL1 sample, but it is still less organized and has less fibrillar structure compared to the native collagen sample. The film remains clumped and aggregated, yet discernible fibrillar structures are apparent, as the profile demonstrates. Notably, the estimated roughness, measured as $R_{a}=1.3\;nm$ aligns more closely with native collagen and has a higher roughness than the GL1 sample. Also, the network looks denser compared to the GL1 sample, but less dense than the native collagen sample. Fibril dimensions range roughly to 4 nm, and their features are larger and more defined than those of the GL1 sample, suggesting a better degree of fibril formation. However, they are still less organized than the fibrils of native collagen.

## 4. Discussion

This study reveals a dual mechanism of adhesion in adipose-derived mesenchymal stem cells (ADMSCs) interacting with collagen substrates, particularly under conditions of glycation. Our data support the existence of two temporally distinct adhesion pathways: a rapid, RAGE-mediated mechanism active within the first 30 min, and a slower, integrin-dependent process that dominates at later stages. This duality is consistent with findings of Olson et al., 2021 [44], who demonstrated that advanced glycation end-products (AGEs) disrupt integrin signaling while promoting RAGE-dependent adhesion and differentiation in myoblasts.

Emerging evidence underscores the pivotal role of the YAP/TAZ signaling axis as a central intracellular mechanism governing cellular behavior in response to mechanical stimuli [26,27]. The activity of this pathway is tightly regulated by the Hippo signaling cascade. When the Hippo pathway is active, YAP/TAZ undergo phosphorylation, resulting in their cytoplasmic retention and subsequent proteasomal degradation [3,45], reflected by a lower nuclear to cytosolic ratio. In contrast, suppression of Hippo signaling permits the nuclear translocation of unphosphorylated YAP/TAZ, where they associate with TEA domain (TEAD1–4) transcription factors to drive the expression of genes involved in proliferation, survival, and differentiation [46]. The temporal overlap between RAGE engagement and YAP/TAZ activation suggests a functional link between these signaling axes. In our study, YAP/TAZ nuclear localization peaked during early adhesion (30 min), coinciding with enhanced attachment to glycated collagen. This activity diminished at later time points (2–5 h), when focal adhesions matured and integrin signaling became dominant. Ref. [47] similarly reported that YAP/TAZ activity in mesenchymal stem cells is tightly regulated by ECM composition and mechanical cues, with collagen deposition enhancing integrin-mediated mechanotransduction. Our findings extend this concept by suggesting that RAGE signaling may serve as an alternative mechanosensitive pathway during early adhesion events.

Glycation of collagen significantly altered the adhesion profile of ADMSCs. Glycated substrates accelerated early adhesion, likely via RAGE signaling, while attenuating focal adhesion formation, as evidenced by reduced vinculin-positive structures. These observations align with the work of Lucas Olson and colleagues [44], who showed that AGE-modified collagen suppresses integrin expression and cytoskeletal organization, while RAGE agonists such as S100b can restore adhesion and differentiation. The attenuation of integrin-mediated adhesion on glycated substrates may reflect structural changes in the ECM that impair classical adhesion receptor engagement.

Interestingly, endogenous collagen synthesis, quantified via immunofluorescence, appeared to be a constitutive process, active during both early and late adhesion phases. As shown in Figure 9, the intensity of the endogenous collagen signal increased approximately twofold with prolonged incubation, reaching a comparable ratio under both native and glycated conditions, and appearing to peak around the 2 h mark. Notably, GL5 consistently displayed higher fluorescence than GL1, with nearly double the signal at 30 min and a sustained, though attenuated, difference at 2 and 5 h. These findings indicate that while endogenous collagen synthesis proceeds in a largely constitutive manner, it remains responsive to the glycation status of the surrounding matrix, particularly under highly glycated conditions such as those in Gl5. Its expression did not correlate directly with YAP/TAZ activity, suggesting that collagen production in ADMSCs may be regulated independently of Hippo pathway dynamics. This biphasic response may reflect differential cellular adaptation to substrate integrity and mechanical feedback.

AFM analysis showed that glycation disrupted collagen self-assembly, with GL1 forming sparse, disorganized structures and GL5 displaying partial fibril restoration through increased aggregation. This improvement after 5 days is likely due to progressive formation of advanced glycation end-products and crosslinks, which stabilize collagen fibrils and modulate adhesion dynamics, consistent with our previous findings [10].

## 5. Conclusions

Taken together, our findings suggest that ADMSC adhesion to glycated collagen involves a dual mechanism: early RAGE-mediated attachment with transient YAP/TAZ activation, followed by integrin-dependent focal adhesion at later stages. Glycation accelerates initial adhesion but impairs integrin signaling, while endogenous collagen synthesis remains constitutive yet responsive to matrix glycation. These findings highlight RAGE as an alternative mechanosensitive pathway and underscore how ECM glycation, characteristic of diabetic and aged tissues, alters stem cell adhesion and signaling. This has important implications for understanding impaired regeneration in pathological microenvironments and for designing biomaterials that better mimic native tissue conditions.

## Figures and Tables

**Table 1:** Morphometric quantification of ADMSC spreading efficiency on native (Nat) and glycated collagen substrates (GL1 and GL5). The analysis was conducted at early (30 min) and later (2 and 5 h) adhesion time points. Cells were fixed and stained for actin, vinculin, and nuclei, as in Figure 1. Quantitative morphometric parameters—including mean Cell Spreading Area (CSA, μm^2^), Perimeter (μm), and Cell Shape Index (CSI)—were calculated using ImageJ 1.54g software.

Parameter	Incubation Time	Nat	GL1	GL5
CSA (μm^2^)	30 min	3165 ± 1926 (n = 11)	1337 ± 762 (n = 11)	1326 ± 907 (n = 14)
	2 h	7522 ± 3767 (n = 20)	5939 ± 4382 (n = 9)	5454 ± 2717 (n = 12)
	5 h	7097 ± 3814 (n = 11)	6663 ± 2440 (n = 12)	6508 ± 2037 (n = 14)
Perimeter (μm)	30 min	225 ± 67	144 ± 48	136 ± 55
	2 h	468 ± 206	409 ± 216	544 ± 198
	5 h	607 ± 220	724 ± 184	854 ± 343
CSI	30 min	0.73 ± 0.18	0.79 ± 0.14	0.87 ± 0.13
	2 h	0.48 ± 0.17	0.56 ± 0.18	0.38 ± 0.12
	5 h	0.28 ± 0.17	0.23 ± 0.08	0.17 ± 0.07

**Table 2:** Quantification of focal adhesion dynamics at 30 min and 2 h of incubation.

Incubation Time	Substrate Type	Number of FAs per Cell	Total Area of FAs per Cell
30 min	Nat	1 ± 1	3.57 ± 0.39 µm^2^
30 min	GL1	2 ± 1	15.14 ± 7.65 µm^2^
30 min	GL5	2 ± 2	15.75 ± 5.04 µm^2^
2 h	Nat	98 ± 29	1407.46 ± 542.01 µm^2^
2 h	GL1	60 ± 12	782.08 ± 267.41 µm^2^
2 h	GL5	51 ± 16	492.60 ± 102.39 µm^2^

## Data Availability

The original contributions presented in this study are included in the article. Further inquiries can be directed to the corresponding author.

## Abbreviations

AD-MSCs	Adipose-Derived Mesenchymal Stem Cells
CAR	Cell Aspect Ratio
CSA	Cell Spreading Area
CSI	Cell Shape Index
ECM	Extracellular Matrix
FBS	Fetal Bovine Serum
GL1	1 day glycated RTC
GL5	5 day glycated RTC
MSCs	Mesenchymal Stem Cells
PBS	Phosphate-Buffered Saline
RAGE	Receptor for Advanced Glycation End-products
RTC	Rat Tail Collagen
TAZ	Transcriptional Co-activator with PDZ-binding Motif
YAP	Yes-Associated Protein

## References

[1] Romani P., Brian I., Santinon G., Pocaterra A., Audano M., Pedretti S., Mathieu S., Forcato M., Bicciato S., Manneville J.B. (2019). Extracellular matrix mechanical cues regulate lipid metabolism through Lipin-1 and SREBP. Nat. Cell Biol..

[2] Raman N., Imran S.A.M., Ahmad Amin Noordin K.B., Wan Kamarul Zaman W.S., Nordin F. (2022). Mechanotransduction in Mesenchymal Stem Cells (MSCs) Differentiation: A Review. Int. J. Mol. Sci..

[3] Humphrey J.D., Dufresne E.R., Schwartz M.A. (2014). Mechanotransduction and extracellular matrix homeostasis. Nat. Rev. Mol. Cell Biol..

[4] Mierke C.T. (2024). Extracellular Matrix Cues Regulate Mechanosensing and Mechanotransduction of Cancer Cells. Cells.

[5] Vining K.H., Mooney D.J. (2017). Mechanical forces direct stem cell behavior in development and regeneration. Nat. Rev. Mol. Cell Biol..

[6] Klein E.A., Yin L., Kothapalli D., Castagnino P., Byfield F.J., Xu T., Levental I., Hawthorne E., Janmey P.A., Assoian R.K. (2009). Cell-cycle control by physiological matrix elasticity and in vivo tissue stiffening. Curr. Biol..

[7] Wang L., You X., Zhang L., Zhang C., Zou W. (2022). Mechanical regulation of bone remodeling. Bone Res..

[8] Kozlova I., Sytnyk V. (2023). Cell Adhesion Molecules as Modulators of the Epidermal Growth Factor Receptor. Cells.

[9] Li D., Zhou J., Chowdhury F., Cheng J., Wang N., Wang F. (2011). Role of mechanical factors in fate decisions of stem cells. Regen. Med..

[10] Komsa-Penkova R., Todinova S., Ivanova V., Stoycheva S., Temnishki P., Dimitrov B., Dimitrov D., Tonchev P., Georgieva G., Kukov A. (2024). Adhesion of Mesenchymal Stem Cells to Glycated Collagen—Comparative Analysis of Dynamic and Static Conditions. Polymers.

[11] Majhy B., Priyadarshinia P., Sen A.K. (2021). Effect of surface energy and roughness on cell adhesion and growth—Facile surface modification for enhanced cell culture. RSC Adv..

[12] Mouw J.K., Ou G., Weaver V.M. (2014). Extracellular matrix assembly: A multiscale deconstruction. Nat. Rev. Mol. Cell Biol..

[13] Heino J. (2014). Cellular signaling by collagen-binding integrins. Adv. Exp. Med. Biol..

[14] Zeltz C., Gullberg D.J. (2016). The integrin-collagen connection—A glue for tissue repair?. Cell Sci..

[15] Komsa-Penkova R., Dimitrov B., Todinova S., Ivanova V., Stoycheva S., Temnishki P., Georgieva G., Tonchev P., Iliev M., Altankov G. (2023). Early Stages of Ex Vivo Collagen Glycation Disrupt the Cellular Interaction and Its Remodeling by Mesenchymal Stem Cells—Morphological and Biochemical Evidence. Int. J. Mol. Sci..

[16] Myllyharju J., Brinckmann J., Notbohm H., Müller P.K. (2005). Intracellular Post-Translational Modifications of Collagens.

[17] Komsa-Penkova R., Yordanova A., Tonchev P., Kyurkchiev S., Todinova S., Strijkova V., Iliev M., Dimitrov B., Altankov G. (2023). Altered Mesenchymal Stem Cells Mechanotransduction from Oxidized Collagen: Morphological and Biophysical Observations. Int. J. Mol. Sci..

[18] Mikulíková K., Eckhardt A., Pataridis S., Mikšík I. (2007). Study of Posttranslational Non-Enzymatic Modifications of Collagen Using Capillary Electrophoresis/Mass Spectrometry and High Performance Liquid Chromatography/Mass Spectrometry. J. Chromatogr. A.

[19] Li Z., Lee H., Zhu C. (2016). Molecular mechanisms of mechanotransduction in integrin-mediated cell-matrix adhesion. Exp. Cell Res..

[20] Lee J.H., Park H.K., Kim K.S. (2016). Intrinsic and extrinsic mechanical properties related to the differentiation of mesenchymal stem cells. Biochem. Biophys. Res. Commun..

[21] Sun Z., Guo S.S., Fässler R. (2016). Integrin-mediated mechanotransduction. J. Cell Biol..

[22] Horbett T.A. (1994). The role of adsorbed proteins in animal cell adhesion. Colloids Surf. B Biointerfaces.

[23] Burridge K., Monaghan Benson E., Graham D.M. (2019). Mechanotransduction: From the cell surface to the nucleus via RhoA. Philos. Trans. R. Soc..

[24] Pang X., He X., Qiu Z., Zhang H., Xie R., Liu Z., Gu Y., Zhao N., Xiang Q., Cui Y. (2023). Targeting integrin pathways: Mechanisms and advances in therapy. Signal Transduct. Target. Ther..

[25] Meng Z., Moroishi T., Guan K. (2016). Mechanisms of Hippo pathway regulation. Genes Dev..

[26] Dupont S., Morsut L., Aragona M., Enzo E., Giulitti S., Cordenonsi M., Zanconato F., Le Digabel J., Forcato M., Bicciato S. (2011). Role of YAP/TAZ in mechanotransduction. Nature.

[27] Zinatizadeh M., Miri S.R., Zarandi P.K., Chalbatani G.M., Rapôso C., Mirzaei H.R., Akbari M.E., Mahmoodzadeh H. (2019). The Hippo Tumor Suppressor Pathway (YAP/TAZ/TEAD/MST/LATS) and EGFR-RAS-RAF-MEK in cancer metastasis. Genes Dis..

[28] Halder G., Dupont S., Piccolo S. (2012). Transduction of mechanical and cytoskeletal cues by YAP and TAZ. Nat. Rev. Mol. Cell Biol..

[29] Pobbati A., Hong W. (2020). A combat with the YAP/TAZ-TEAD oncoproteins for cancer therapy. Theranostics.

[30] Komsa-Penkova R., Stavreva G., Belemezova K., Kyurkchiev S., Todinova S., Altankov G. (2022). Mesenchymal Stem-Cell Remodeling of Adsorbed Type-I Collagen-The Effect of Collagen Oxidation. Int. J. Mol. Sci..

[31] Komsa-Penkova R., Spirova R., Bechev B. (1996). Modification of Lowrys Method for Collagen Concentration Measurement. J. Biochem. Biophys. Methods.

[32] Kuznetsova N., Leikin S. (1999). Does the Triple Helical Domain of Type I Collagen Encode Molecular Recognition and Fiber Assembly While Telopeptides Serve as Catalytic Domains. J. Biol. Chem..

[33] McQuin C., Goodman A., Chernyshev V., Kamentsky L., Cimini B.A., Karhohs K.W., Carpenter A.E. (2018). CellProfiler 3.0: Next-generation image processing for biology. PLOS Biol..

[34] Stirling D.R., Carpenter A.E., Cimini B.A. (2021). CellProfiler Analyst 3.0: Accessible data exploration and machine learning for image analysis. Bioinformatics.

[35] Boudaoud A., Burian A., Borowska-Wykręt D., Uyttewaal M., Wrzalik R., Kwiatkowska D., Hamant O. (2014). FibrilTool, an ImageJ Plug-in to Quantify Fibrillar Structures in Raw Microscopy Images. Nat. Protoc..

[36] Antonio P.D., Lasalvia M., Perna G., Capozzi V. (2012). Scale-independent roughness value of cell membranes studied by means of AFM technique. Biochim. Biophys. Acta.

[37] Sessa L., Gatti E., Zeni F., Antonelli A., Catucci A., Koch M., Pompilio G., Fritz G., Raucci A., Bianchi M.E. (2014). The Receptor for Advanced Glycation End-Products (RAGE) Is Only Present in Mammals, and Belongs to a Family of Cell Adhesion Molecules (CAMs). PLoS ONE.

[38] Ramasamy R., Yan S.F., Schmidt A.M. (2011). Receptor for AGE (RAGE): Signaling mechanisms in the pathogenesis of diabetes and its complications. Ann. N. Y. Acad. Sci..

[39] Murillo J., Wang Y., Xu X., Klebe R.J., Chen Z., Zardeneta G., Pal S., Mikhailova M., Steffensen B. (2008). Advanced glycation of type I collagen and fibronectin modifies periodontal cell behavior. J. Periodontol..

[40] Dandia H., Makkad K., Tayalia P. (2019). Glycated collagen—A 3D matrix system to study pathological cell behavior. Biomater. Sci..

[41] Crop M.J., Baan C.C., Korevaar S.S., IJzermans J.N.M., Pescatori M., Stubbs A.P., Van IJcken W.F.J., Dahlke M.H., Eggenhofer E., Weimar W. (2010). Inflammatory conditions affect gene expression and function of human adipose tissue-derived mesenchymal stem cells. Clin. Exp. Immunol..

[42] Franz C.M., Muller D.J., Braga P., Ricci D. (2011). Studying Collagen Self-Assembly by Time-Lapse High-Resolution Atomic Force Microscopy. Atomic Force Microscopy in Biomedical Research; Methods in Molecular Biology.

[43] Sloseris D., Forde N.R. (2025). Ageing of collagen: The effects of glycation on collagen’s stability, mechanics and assembly. Matrix Biol..

[44] Olson L.C., Nguyen T.M., Heise R.L., Boyan B.D., Schwartz Z., McClure M.J. (2021). Advanced Glycation End Products Are Retained in Decellularized Muscle Matrix Derived from Aged Skeletal Muscle. Int. J. Mol. Sci..

[45] Johnson R., Halder G. (2014). The two faces of Hippo: Targeting the Hippo pathway for regenerative medicine and cancer treatment. Nat. Rev. Drug Discov..

[46] Janmey P.A., Wells R.G., Assoian R.K., McCulloch C.A. (2013). From tissue mechanics to transcription factors. Differentiation.

[47] Komatsu N., Kajiya M., Motoike S., Takewaki M., Horikoshi S., Iwata T., Ouhara K., Takeda K., Matsuda S., Fujita T. (2018). Type I collagen deposition via osteoinduction ameliorates YAP/TAZ activity in 3D floating culture clumps of mesenchymal stem cell/extracellular matrix complexes. Stem Cell Res. Ther..

